# Impaired brain glucose metabolism and presynaptic dopaminergic functioning in a mouse model of schizophrenia

**DOI:** 10.1186/s13550-020-00629-x

**Published:** 2020-04-17

**Authors:** Eugenia Tomasella, German Falasco, Leandro Urrutia, Lucila Bechelli, Lucia Padilla, Diego M. Gelman

**Affiliations:** 1 Instituto de Biología y Medicina Experimental, Consejo Nacional de Investigaciones Científicas y Técnicas, Vuelta de Obligado 2490, C1428ADN Ciudad de Buenos Aires, Argentina; 2 Fleni, Centro de Imágenes Moleculares (CIM), Laboratorio de Imágenes Preclínicas, Buenos Aires, Argentina

**Keywords:** Schizophrenia, Mouse model, PET, Glucose metabolism, [^18^F]-FDG, Dopamine, [^18^F]-F-DOPA

## Abstract

**Background:**

Schizophrenia is a disease diagnosed by visible signs and symptoms from late adolescence to early adulthood. The etiology of this disease remains unknown. An objective diagnostic approach is required. Here, we used a mouse model that shows schizophrenia-like phenotypes to study brain glucose metabolism and presynaptic dopaminergic functioning by positron emission tomography (PET) and immunohistochemistry. PET scannings were performed on mice after the administration of [^18^F]-FDG or [^18^F]-F-DOPA. Glucose metabolism was evaluated in basal conditions and after the induction of a hyperdopaminergic state.

**Results:**

Mutant animals show reduced glucose metabolism in prefrontal cortex, amygdala, and nucleus reuniens under the hyperdopaminergic state. They also show reduced [^18^F]-F-DOPA uptake in prefrontal cortex, substantia nigra reticulata, raphe nucleus, and ventral striatum but increased [^18^F]-F-DOPA uptake in dorsal striatum. Mutant animals also show reduced tyrosine hydroxylase expression on midbrain neurons.

**Conclusions:**

Dopamine D2 mutant animals show reduced glucose metabolism and impaired presynaptic dopaminergic functioning, in line with reports from human studies. This mouse line may be a valuable model of schizophrenia, useful to test novel tracers for PET scanning diagnostic.

## Background

Schizophrenia is a syndrome of still unknown etiology that affects 1% of world population. It is diagnosed between late adolescence and early adulthood only by visible signs and symptoms [1]. Among them, the most characteristics are hallucinations and delusions that emerge in the context of a psychotic break. These symptoms are well treated by the administration of antipsychotics, which are antagonists of dopamine D2 receptors (DRD2) [2]. However, other symptoms of the disease do not improve after antipsychotic administration, like low mood, apathy, anhedonia, and cognitive impairments and, therefore, a specific pharmacological approach is required.

The dopaminergic hypothesis of schizophrenia is the most perdurable in the field. It postulates an abnormal dopaminergic neurotransmission with an imbalance between cortical and subcortical regions. A reduction in dopamine tone in prefrontal cortex is accompanied by an increased dopamine tone in the striatum, underling negative and positive symptoms of the disease, respectively [3]. In this context, antipsychotic blockade of D2 receptors in the striatum might prevent excessive DRD2 signaling. Post-mortem studies from schizophrenia patients have shown a reduced expression of the rate-limiting enzyme of dopamine synthesis, tyrosine hydroxylase (TH) in midbrain dopaminergic neurons [4]. This observation was associated to the prefrontal hypodopaminergic state. However, there is no conclusive evidence of dysfunction within the dopaminergic system itself in schizophrenia although increased dopamine signaling is a common finding in schizophrenia patients. Instead, research efforts are focused on the regulation of dopamine release from midbrain neurons modulated by afferent structures like the ventral subiculum [5], pedunculopontine tegmentum [6], or the nucleus reuniens [7, 8]. Alternatively, the GABAergic hypothesis of the disease postulates an impaired development of inhibitory neurons that results in an aberrant excitatory/inhibitory balance. Interestingly, among the population of inhibitory neurons, those expressing the calcium-binding protein parvalbumin seem to be particularly affected [9–11].

The generation of animal models by neonatal lesions, pharmacological or genetic tools proved to be highly valuable in the way to understand the origin of schizophrenia [12–15]. In view of the aforementioned hypothesis of schizophrenia, we generated a mice line with a selective deletion of DRD2 exclusively from parvalbumin interneurons [16]. DRD2 mutant animals show adult onset of behaviors reminiscent of schizophrenia and endophenotypes at molecular, cellular, and physiological levels similar to those described from patients studies [16]. As the selective deletion of DRD2 from parvalbumin interneurons reproduces a number of phenotypes found in patients, we decided to further characterize this mouse line.

Positron emission tomography (PET) is a versatile, non-invasive technique that detects tissue distribution of specific labeled tracers. Among the most used tracer in clinical diagnostics is the fluorinated glucose analog [^18^F]-FDG, which localizes in metabolically active tissues and accumulates in an activity-dependent manner. Many reports have shown reduced glucose metabolic rate in the frontal cortex of patients with schizophrenia, i.e., hypofrontality by [18F]-FDG PET scanning [17–20]. Presynaptic dopaminergic functioning may be also studied by a PET scanner in vivo by the administration of [^18^F]-F-DOPA (Fluorodopa), as it is incorporated by presynaptic monoaminergic neurons, decarboxylated to [^18^F]-fluorodopamine by the aromatic amino acid decarboxylase (AADC) and then stored in vesicles. The conversion of [^18^F]-F-DOPA to [^18^F]-fluorodopamine is useful to estimate the dopamine synthesis capacity and vesicular storage of monoaminergic neurons. It has been shown that schizophrenia patients have an elevated striatal presynaptic dopamine synthesis capacity by [^18^F]-F-DOPA PET scanning experiments [21–23].

In the clinic, psychiatric patients show different characteristics associated to the phase of the illness, pharmacological treatment, environmental context, weight, sex or age, among others, making the analysis of PET studies variable and discrepant. As a consequence, PET scanning with [^18^F]-FDG, [^18^F]-F-DOPA, or other tracers, is not a routine imaging study to assist in the diagnosis and/or evolution of psychiatric diseases.

In this work, we took advantage of the mutant DRD2 mouse model [16] to perform preclinical PET experiments in a genetically, physiologically, and environmentally homogeneous animal population, naïve of any antipsychotic treatment, to assess glucose metabolism, presynaptic dopaminergic functioning, and tyrosine hydroxylase expression. We tested the hypothesis that mutant animals have reduced glucose metabolic consumption in the prefrontal cortex and abnormal uptake of [^18^F]-F-DOPA compared to control animals. Second, we investigated tyrosine hydroxylase in substantia nigra (SN) and ventral tegmental area (VTA) to identify changes in the expression level.

## Methods

### Animal model

To generate a selective DRD2 deletion from parvalbumin interneurons, a Parvalbumin-Cre line [24] was mated to a floxed DRD2 line [25] in a C57BL6 background. Genotyping for the Cre gene and floxed alleles was performed as previously described [16]. Animals with DRD2^flox/flox^ or Pvalb-Cre::DRD2^flox/flox^ genotype were used as control or mutant groups, respectively. Animals were between 80 and 90 days old at the first [^18^F]-FDG scanning and all experiments were performed with male mice.

#### Imaging system

Images were acquired using a preclinical PET TriFoil Lab-PET 4 with an approximated spatial resolution of 1.2 mm (full width at half maximum) and 3.7 cm axial and 11 cm trans-axial FOV (field of view).

#### Animal procedures

Two groups of adult animals (*n* = 10 controls and *n* = 10 mutants) were used for PET scanning experiments. Both groups were studied with [^18^F]-FDG and [^18^F]-F-DOPA. The first experiment was the [^18^F]-FDG in basal conditions. The next week, the [^18^F]-FDG with amphetamine experiment were performed and 1 week later the [^18^F]-F-DOPA experiment. During the scanning, mice were anesthetized using a mixture of isoflurane and O_2_ (inhalation, 4.5% induction and 1.5% maintenance dose) and maintained in a warm table (35 °C).

For [^18^F]-FDG acquisitions, adult animals were starved during 4 h and then injected with 0.925 MBq/gr i.p. and left undisturbed in an individual temperature-controlled (29 °C) cage for 30 min during radiopharmaceutical incorporation. Each subject was acquired for 12 min using list-mode acquisition. [^18^F]-FDG experiment was performed again a week later on both groups under the same conditions but in this setup 5 mg/kg of amphetamine (i.p.) (Sigma) were administered 15 min before the [^18^F]-FDG administration in order to induce a massive dopamine outflow.

One week after the second [^18^F]-FDG acquisition, the two groups of animals were injected with 3.7 MBq/gr i.v. of [^18^F]-F-DOPA, 30 min after the preadministration of carbidopa (10 mg/kg, i.p.), and left undisturbed for 80 min during radiopharmaceutical incorporation. Then, each subject was acquired for 30 min using list-mode acquisition.

#### Imaging reconstruction

Images were reconstructed using an OSEM 3D algorithm with 30 iterations, to maximize SNR (signal-to-noise ratio). If motion was detected during acquisition, a dynamic reconstruction was performed in order to correct it using SPM8 on MATLAB® realign algorithm.

#### Spatial image processing

A previously generated normal [^18^F]-FDG template was used in order to have an anatomic reference for realignment and normalization. [^18^F]-FDG images were normalized to the template using SPM8 on MATLAB® (normalized mutual information as objective function and 7-mm smoothing histogram for rigid co-registration and affine regularization to the averaged template size, no-smooth and 2–0.1 mm of separation for the non-rigid normalization). All images were smoothened using an isotropic Gaussian kernel with 1 mm FWHM. [^18^F]-F-DOPA images were previously co-registered to the [^18^F]-FDG for each subject and transformation resultant from each [^18^F]-FDG normalization was applied to co-registred [^18^F]-F-DOPA images.

Intensity normalization of [^18^F]-FDG images were referenced to gray cerebellum and [^18^F]-F-DOPA to all brain uptakes. A brain masking avoiding Harderian glands was used for [^18^F]-FDG since the uptake in these glands is too variable.

#### Image statistical analysis

For [^18^F]-FDG, analyzed groups were as follows: control in basal conditions, control after amphetamine treatment, mutant in basal condition, and mutant after amphetamine treatment. For [^18^F]-F-DOPA, analyzed groups were control and mutant animals in basal conditions. All subject groups were analyzed as a full-factorial ANOVA test using SPM8 on MATLAB®. Intensity normalization was considered as a regressor variable for each factor using grand mean scaling (ANCOVA). Global calculation of individual means was calculated over each masked brain. Parametric statistical images were calculated for group’s contrasts: control basal vs. mutant basal, control basal vs. control amphetamine, mutant basal vs. mutant amphetamine, control amphetamine vs. mutant amphetamine in the [^18^F]-FDG experiments, and control vs. mutant in the [^18^F]-F-DOPA experiment. In order to correct for multiple comparisons, false discovery rate (FDR) approach was applied using SPM8 (*p* value FDR 0.05). In order to have an accurate anatomical reference, all results of statistical differences where co-registered with an MRI atlas. Spatial transformation was applied to the MRI atlas to correct for the differences between mice strains and methodological animal handling.

#### Immunohistochemistry and image analysis

Mice were transcardially perfused with 4% paraformaldehyde (PFA) and the brain was removed and postfixed in the same fixative for 180 min at 4 °C. The tissue was cryoprotected sequentially in 10%, 20%, and 30% sucrose solution in phosphate buffer saline (PBS) and then cut serially in a cryostat in 40 μm thick coronal brain sections. Sections were incubated 1 h in 1% H_2_O_2_ in PBS to inactivate endogenous peroxidases and then rinsed in PBS. A rabbit polyclonal anti-tyrosine hydroxylase antibody was used at 1:1000 (Millipore, AB152). Sections were incubated overnight at 4 °C in PBS, 0.25% Triton X-100, 2% BSA, and 10% NGS (Normal Goat Serum, Natocor). After washing twice for 10 min with PBS, sections were incubated with a solution containing a goat anti-rabbit IgG-peroxidase 1:500 (Vector Laboratories; PI-1000) in the same solution as described previously for 2 h and then rinsed twice 10 min in PBS. Finally, sections were incubated in a solution of 0.025% diaminobenzidine (DAB), 0.05% H_2_O_2_ in TBS (150 mm NaCl; 50 mm tris-HCl; pH 7.2). Immunohistochemistry in sections of control and mutant mice (*n* = 3 for each genotype) were performed in parallel with the same solutions and equal developing time, preventing signal saturation. Images were acquired using an Olympus IX83 microscope. DAB intensity in control and mutant SN (substantia nigra)/VTA (ventral tegmental area) regions were assessed by digital quantification to avoid observer bias using Image J color deconvolution plug-in [26, 27]. Signal intensity was analyzed by *t* test.

## Results

### [^18^F]-FDG uptake analysis

**Fig. 1:**
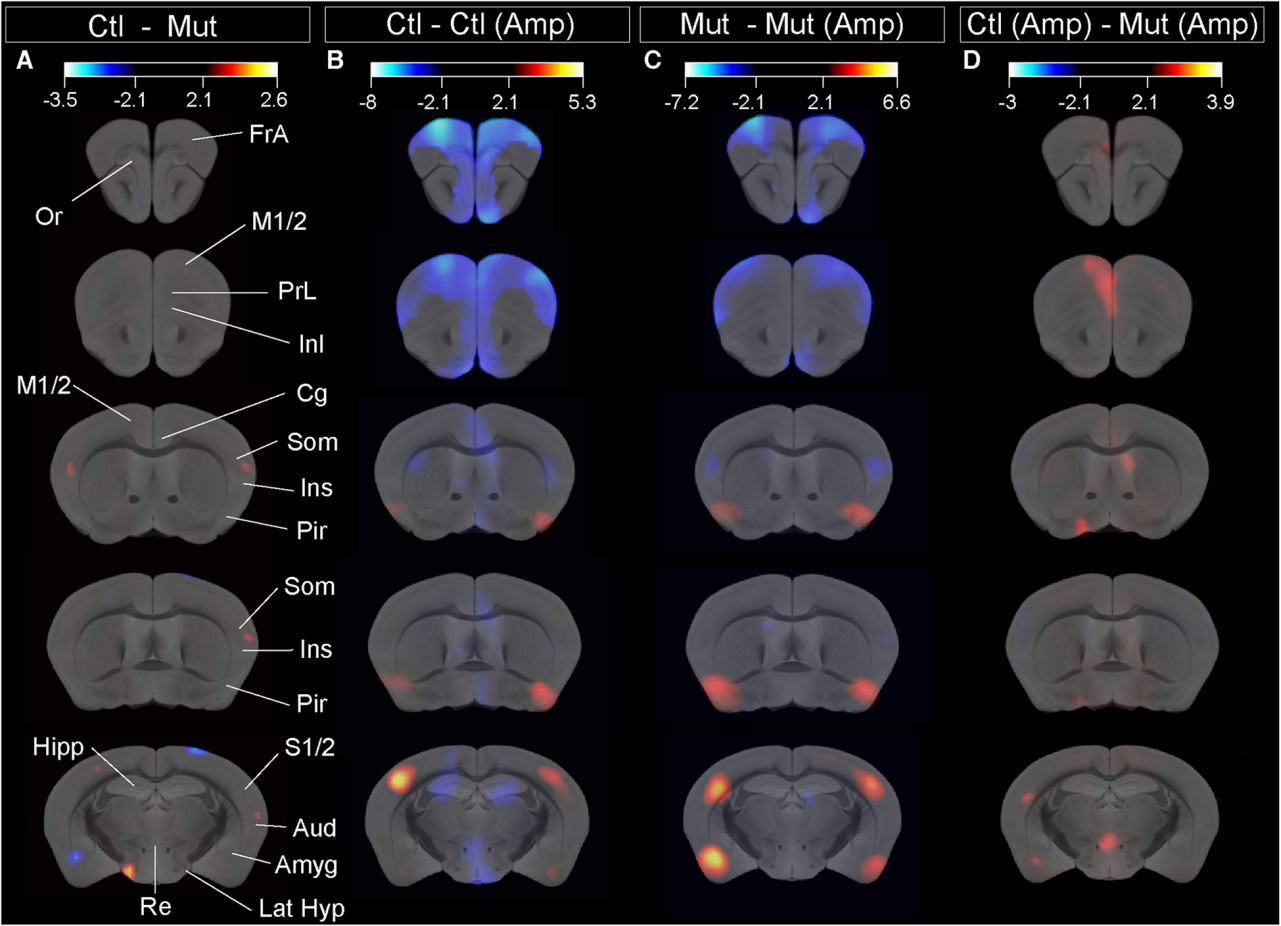
[^18^F]-FDG PET scanning in control and D2 mutant animals under basal conditions or after the induction of a hyperdopaminergic state. Subtractive analysis of [^18^F]-FDG incorporation in the following: **a** control and mutant animals in basal conditions. **b** Control animals in basal condition and control animals in amphetamine-treated condition. **c** Mutant animals in basal condition and amphetamine-treated condition. **d** Control and mutant animals in amphetamine-treated conditions. [^18^F]-FDG incorporation is depicted in red-yellow color for metabolic activity increase in control animals (**a**), animals in basal conditions (**b**, **c**), or control animal after amphetamine treatment (**d**) or blue-white color for metabolic activity increase in mutant animals in basal condition (**a**) or animals after amphetamine treatment (**b**–**d**). *t* values for *p* < 0.05 are specified for each comparison. FrA frontal association cortex, M1/2 primary/secondary motor cortex, Or orbital cortex, PrL prelimbic cortex, InL infralimbic cortex, Cg cingulate cortex, Som somatosensory cortex, Ins insular cortex, Pir piriform cortex, Aud auditory cortex, Hipp hippocampus, Amyg amygdala, Re reuniens nucleus, Lat Hyp lateral hypothalamus

Local [^18^F]-FDG brain uptake differences between control and mutant animal groups in basal conditions show that control animals have an increased glucose metabolism in the somatosensory/insular cortex and lateral hypothalamic area (Fig. 1a; red-yellow color) and mutant animals show an increased metabolic rate in the basolateral amygdala (Fig. 1a; blue-white color).

We expected wider metabolic differences between groups, especially in prefrontal cortex, but found a significant change restricted to the somatosensory/insular cortex and basolateral amygdala. As the dopaminergic hypothesis of schizophrenia postulates an increased dopaminergic tone underling psychosis [3], we administered amphetamine (5 mg/kg; i.p.) to animals of both genotypes to induce massive dopamine outflow to evaluate glucose metabolic consumption under these conditions and performed a new [^18^F]-FDG PET scanning. Then, we performed a voxel-wise statistical analysis in basal and induced conditions between all groups.

[^18^F]-FDG brain uptake between control animals in basal conditions and control animals after amphetamine treatment shows that the frontal cortex, including the frontal association cortex, primary and secondary motor cortex, prelimbic, infralimbic, orbital, and cingulate cortex as well as the dorsal hippocampus, nucleus reuniens, and hypothalamus exhibit an increased metabolic rate in amphetamine-treated control animals compared to naïve control animals (Fig. 1b; blue-white color). However, the piriform and somatosensory cortex of non-treated animals show increased metabolic rate compared to those that received amphetamine (Fig. 1b; red-yellow color).

Amphetamine-treated mutant animals show increased glucose metabolic rates compared to naïve mutant animals in the frontal associative cortex, secondary motor cortex, and rostral somatosensory cortex but not in the prelimbic, infralimbic, orbital, and cingulate cortex (Fig. 1c; blue-white color). Naïve mutant animals show increased glucose metabolic rate in the piriform cortex and somatosensory cortex, but not in the hypothalamus (Fig. 1c; red-yellow color). Interestingly, the amygdala of naïve mutant animals show increased glucose metabolic rate relative to amphetamine-treated mutant animals.

Finally, amphetamine-treated mutant animals show reduced glucose metabolism in the orbital cortex, secondary motor cortex, cingulate, prelimbic and infralimbic cortex, somatosensory cortex, amygdala, and nucleus reuniens when compared to control, amphetamine-treated animals (Fig. 1d; red-yellow color).

#### [^18^F]-F-DOPA uptake analysis

**Fig. 2:**
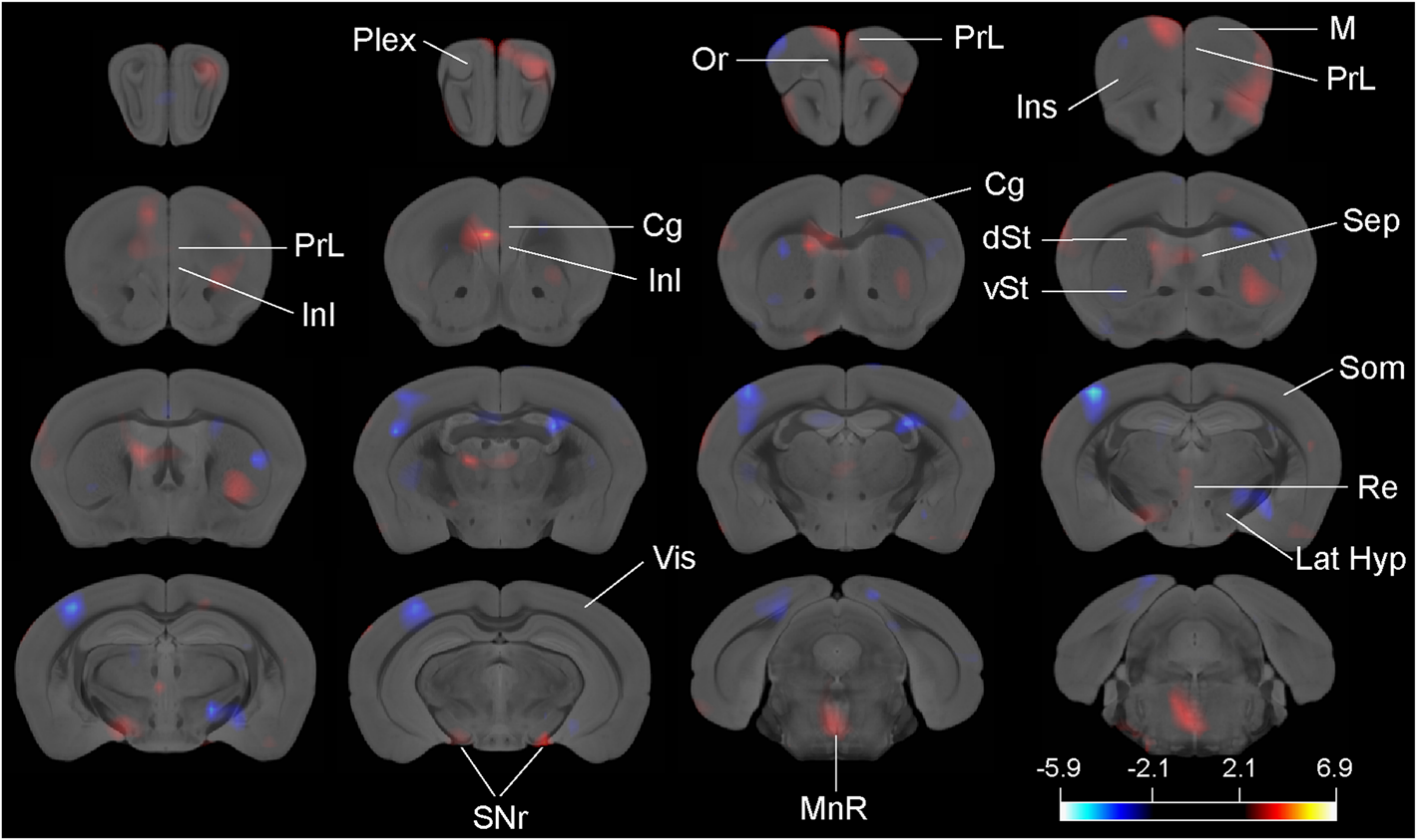
[^18^F]-F-DOPA PET scanning in control and D2 mutant animals. Red-yellow color represents a significant increase of [^18^F]-F-DOPA incorporation in control animals and blue-white color a significant increase of [^18^F]-F-DOPA incorporation in mutant D2 animals. *t* value for *p* < 0.05 is specified. Plex plexiform region, M motor cortex, Or orbital cortex, PrL prelimbic cortex, InL infralimbic cortex, Cg cingulate cortex, Som somatosensory cortex, Ins insular cortex, dSt dorsal striatum, vSt ventral striatum, Sep septum, Re reuniens nucleus, Lat Hyp lateral hypothalamus, Vis visual cortex, SNr substantia nigra reticulata, MnR median raphe

Statistical differences between groups show that mutant D2 animals exhibit reduced [^18^F]-DOPA accumulation in prefrontal cortex, ventral striatum, substantia nigra reticulata, and in the raphe nucleus (Fig. 2; red-yellow color) but an increased uptake in dorsal striatum, somatosensory, and visual cortex (Fig. 2; blue-white color), even in the absence of a hyperdopaminergic state.

### Tyrosine hydroxylase expression in midbrain neurons

**Fig. 3:**
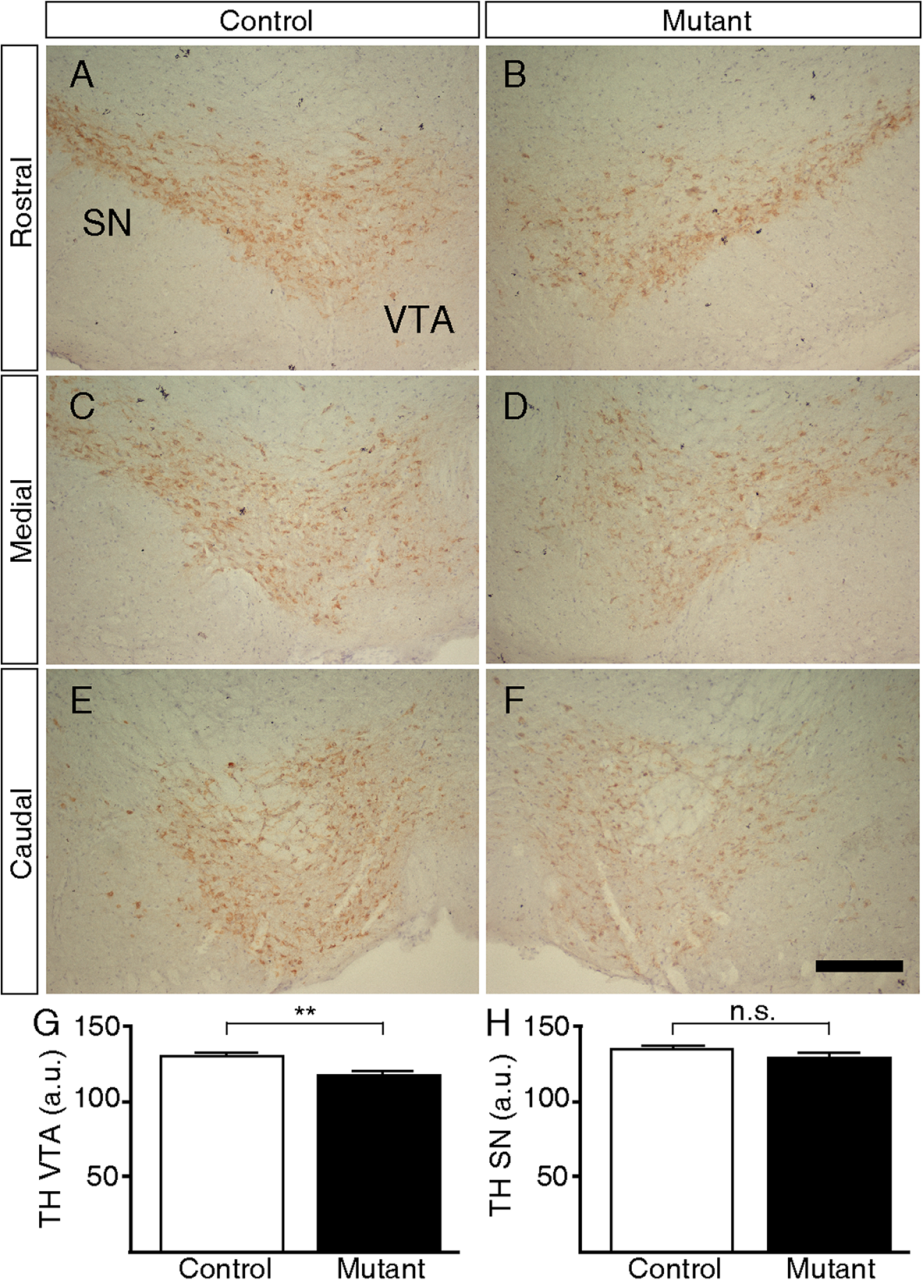
TH expression level assessment by immunohistochemistry. Brain sections spanning rostral (**a**, **b**), medial (**c**, **d**), or caudal (**e**, **f**) sections of the SN/VTA from control (**a**, **c**, **e**) or mutant (**b**, **d**, **f**) animals. Digital quantification of TH expression in the VTA (**g**) and SN (**h**). *t* test, **p < 0,01. n.s. non-significant. All images are × 10 magnifications. SN substantia nigra, VTA ventral tegmental area. Scale bar 200 μm

As our PET studies show reduced [^18^F]-F-DOPA incorporation in mutant animals compared to controls in frontal cortex and other brain areas, we performed immunohistochemical experiments to determine the expression levels of the rate-limiting enzyme of dopamine synthesis, tyrosine hydroxylase (TH). Our results show that TH expression is reduced in the VTA region of mutant animals but not in the substantia nigra compacta. Along the rostro-caudal axis, the expression of TH shows a consistent reduced expression in the VTA region, including the parabrachial pigmented nucleus (PBP), paranigral nucleus of the VTA (PN), and VTA itself (Fig. 3a–f). A quantitative assessment of TH levels shows that the expression is significantly reduced in the VTA of mutant animals but not in the SN (Fig. 3g, h).

## Discussion

In this work, we performed preclinical PET scanning experiments in an animal model of schizophrenia to evaluate brain glucose metabolism and dopamine presynaptic functioning after the administration of [^18^F]-FDG or [^18^F]-F-DOPA, respectively. The voxel-wise statistical analysis between control and mutant animals allowed identifying brain regions that exhibit differential glucose metabolic activity or [^18^F]-F-DOPA uptake.

[^18^F]-FDG experiments show that mutant animals have an impaired glucose metabolism compared to control animals both in basal and hyperdopaminergic conditions. In basal conditions mutant animals show abnormal glucose metabolic activity in the somatosensory/insular cortex, auditory cortex, lateral hypothalamus, and amygdala. However, under a hyperdopaminergic state, mutant animals show a reduced metabolic activity in prefrontal cortex, a pathological condition already described from patient PET studies [18, 20, 28]. Interestingly, in the hyperdopaminergic condition, glucose metabolism is also abnormal in the nucleus reuniens and amygdala, two brain regions involved in the modulation of dopamine release [7, 29]. Our results suggest that the metabolic performance of the prefrontal cortex in mutant animals is affected and the metabolic activity cannot be further increased to compensate the demand imposed by the enhanced dopaminergic tone. In addition, the amygdala and nucleus reuniens follow the same deficient metabolic activity pattern as the prefrontal cortex, highlighting the relevance that these nuclei may have in the disease.

Reduced dopamine D2 receptor availability was associated to a reduction in glucose metabolism [30] and simultaneous voltammetric studies showed an association between dopamine and increased metabolic demand [31]. A recent study in humans showed a linear relationship between dopamine D2/D3 receptor availability and glucose metabolism, suggesting that a hyperdopaminergic state may contribute to the downregulation of dopamine receptors but also to the hypometabolism in unmedicated schizophrenia patients [32]. In our mouse model, we selectively deleted dopamine D2 receptors from parvalbumin interneurons resulting in subcortical hyperdopaminergia, prefrontal hypodopaminergia, and a marked decrease in prefrontal total DRD2 mRNA [16]. Therefore, diminished dopamine D2 receptor availability and reduced dopaminergic neurotransmission in PFC of mutant animals may drive the poor metabolic adaptation observed in the [^18^F]-FDG experiment in the context of hyperdopaminergic state, as reported from individuals with schizophrenia [32].

The dopaminergic hypothesis of schizophrenia postulates an increased presynaptic dopamine functioning in the dorsal striatum [22, 23]. Dopaminergic neurons can incorporate l-DOPA and use it to synthesize dopamine. However, it has been shown that serotonergic neurons also incorporate l-DOPA and then co-release dopamine and serotonin in target regions [33]. l-DOPA administration to rodents produces an increased dopamine accumulation in the prefrontal cortex and substantia nigra reticulata, mainly by the incorporation into serotonergic neurons [34]. Brain metabolism of [^18^F]-F-DOPA is comparable to that of l-DOPA [35]. Taken together, our results point to a regional hypodopaminergic/hyposerotonergic state in mutant animals, as [^18^F]-F-DOPA uptake is reduced in frontal cortex, substantia nigra reticulata, ventral striatum, and raphe nucleus. However, mutant animals show a hyperdopaminergic/hyperserotonergic state in dorsal striatum, visual, and somatosensory cortex due to an increased uptake of [^18^F]-F-DOPA.

Although dysfunction of the dopaminergic system is central in the pathophysiology of schizophrenia, the role of the serotonergic system in the disease is of interest, as most second generation antipsychotics antagonize serotonin receptors. The mesocortical dopaminergic pathway shows a preference to innervate rostral brain regions, and the dopamine D2 receptor distribution and dopamine concentration show the same rostro-caudal gradient. However, the dopaminergic system exhibits minimal innervations of the visual cortex [36–38]. In contrast, the serotonergic system shows a prominent axonal connectivity to sensory areas including visual, auditory, and somatosensory cortex [38]. Our PET experiment shows an increased [^18^F]-F-DOPA uptake in the visual and somatosensory cortex that may be associated to a hyperserotonergic activity. In patients, this exacerbated serotonergic activity may be the substrate of visual and/or auditory hallucinations and may be blocked by the serotonin receptor antagonism activity of second generation antipsychotics.

Midbrain dopaminergic neurons mainly project their axons to the prefrontal cortex. As we observed reduced [^18^F]-F-DOPA uptake in frontal cortex of mutant animals, we performed a quantitative assessment of TH expression in ventral tegmental area (VTA) neurons. It has been reported that the expression of TH, the rate-limiting enzyme of dopamine synthesis, is increased in the substantia nigra (SN) of schizophrenia patients [39]. However, other authors found a reduced expression of TH in the rostral subregion of the ventral tegmental area [4, 40]. These apparent discrepancies may relay on methodological reasons in tissue preparation and immunohistochemistry [4]. Diaminobenzidine developing time was carefully observed in our experiments, as prevention of signal saturation is crucial for accurate quantification [26]. TH expression from midbrain VTA neurons of mutant animals shows a consistent decreased expression, in line with a previous report [4].

The development of mouse models of schizophrenia proved to be useful to understand different aspects of the disease [5, 14, 15]. However, few studies used them to analyze brain metabolic activity or presynaptic dopaminergic function by PET scanning [41]. We previously showed that parvalbumin D2 mutant animals exhibit molecular, cellular, physiological, and behavioral impairments highly reminiscent of symptoms observed in schizophrenia patients and a parallel developmental onset [16]. Here, we provide evidence of impaired brain glucose metabolism, presynaptic dopamine dysfunction, and reduced TH expression, phenotypes that has a correlate with patient studies [4, 17–19, 23, 32].

Different PET tracers are available to evaluate dopamine receptors [42], acetylcholine receptors [43] or glial activation [44] by a non-invasive approach. However, most of them are still under clinical research, as no conclusive evidence supports their use in clinical psychiatric diagnostics. The parvalbumin D2 null mice line (PV^D2null^) may be an interesting preclinical model to test these and other novel PET tracers with potential use in schizophrenia diagnostics. In our [^18^F]-FDG experiments, mutant D2 animals show a parallel pattern of glucose metabolism as patients in the frontal cortex but points to nucleus reuniens and amygdala also playing a critical role in the disease. At the same time, [^18^F]-F-DOPA experiments highlight the relevance that substantia nigra reticulata and raphe nucleus may have in the pathophysiology of schizophrenia. Careful analyses of PET scannings with [^18^F]-FDG and [^18^F]-F-DOPA taking into account these brain regions may lead to accurate results with potential use in psychiatric diagnostics.

## Conclusions

Dopamine D2 receptor mutant mice line may be a suitable schizophrenia model, providing homogenous genetic background and developmental physiological conditions to test novel tracers with the potential to develop a reliable diagnostic imaging method. Finally, it may pave the way to translate preclinical settings into the clinic, expanding the diagnostic potential of PET scanning.

## Data Availability

The datasets used and analyzed during the current study are available from the corresponding author on reasonable request.

## Abbreviations

DRD2: Dopamine D2 receptor
PET: Positron emission tomography
F-DOPA: 6 fluoro-L-3,4-dihydroxyphenylalanine
TH: Tyrosine hydroxylase
VTA: Ventral tegmental area
SN: Substantia nigra
NGS: Normal goat serum
